# Direct Electrochemistry of Hemoglobin Immobilized on a Functionalized Multi-Walled Carbon Nanotubes and Gold Nanoparticles Nanocomplex-Modified Glassy Carbon Electrode

**DOI:** 10.3390/s130708595

**Published:** 2013-07-05

**Authors:** Jun Hong, Ying-Xue Zhao, Bao-Lin Xiao, Ali Akbar Moosavi-Movahedi, Hedayatollah Ghourchian, Nader Sheibani

**Affiliations:** 1 School of Life Sciences, Henan University, JinMing Road, Kaifeng 475000, China; E-Mails: 66yingxue@163.com (Y.-X.Z.); arixxl@163.com (B.-L.X.); 2 Institute of Biochemistry and Biophysics, University of Tehran, Enquelab Avenue, P.O. Box 13145-1384, Tehran, Iran; E-Mail: hadi@ibb.ut.ac.ir; 3 Department of Ophthalmology and Visual Sciences, University of Wisconsin, 600 Highland Avenue, K6/456 CSC, Madison, WI 53792-4673, USA; E-Mail: nsheibanikar@wisc.edu

**Keywords:** hemoglobin, direct electrochemistry, functionalized multi-walled carbon nanotubes, gold nanoparticles, nanocomplex

## Abstract

Direct electron transfer of hemoglobin (Hb) was realized by immobilizing Hb on a carboxyl functionalized multi-walled carbon nanotubes (FMWCNTs) and gold nanoparticles (AuNPs) nanocomplex-modified glassy carbon electrode. The ultraviolet-visible absorption spectrometry (UV-Vis), transmission electron microscopy (TEM) and Fourier transform infrared (FTIR) methods were utilized for additional characterization of the AuNPs and FMWCNTs. The cyclic voltammogram of the modified electrode has a pair of well-defined quasi-reversible redox peaks with a formal potential of −0.270 ± 0.002 V (*vs.* Ag/AgCl) at a scan rate of 0.05 V/s. The heterogeneous electron transfer constant (ks) was evaluated to be 4.0 ± 0.2 s^−1^. The average surface concentration of electro-active Hb on the surface of the modified glassy carbon electrode was calculated to be 6.8 ± 0.3 × 10^−10^ mol cm^−2^. The cathodic peak current of the modified electrode increased linearly with increasing concentration of hydrogen peroxide (from 0.05 nM to 1 nM) with a detection limit of 0.05 ± 0.01 nM. The apparent Michaelis-Menten constant (K_m_^app^) was calculated to be 0.85 ± 0.1 nM. Thus, the modified electrode could be applied as a third generation biosensor with high sensitivity, long-term stability and low detection limit.

## 1. Introduction

Direct electrochemistry of redox proteins immobilized on different electrodes has recently attracted great attention. These methods can provide a suitable model for understanding the electron transfer mechanisms in biological systems and to establish a foundation for fabrication of electrochemical biosensors and devices [1–4]. Successful approaches have included cast films of redox proteins with different materials and membranes [5–23]. Achieving direct electron transfer between redox proteins or enzymes and electrodes simplifies these devices, which has a great significance in preparing the third generation of biosensors [24].

Hemoglobin (Hb) is an oxygen carrier and as a pro-oxidant takes part in complex redox processes in the blood. This protein consists of two alpha and two beta subunits with a molecular weight of about 67,000 and each subunit has one peptide chain and one protoheme [25–27]. Hb structure is similar to the peroxidases and can catalyze the reduction of hydrogen peroxide. [28,29]. Direct electron transfer of Hb immobilized on various electrode materials has become popular. In addition, the structure of Hb is known and can be used as a model to explain the relationship between the protein structure and function and to construct new functional biosensors without mediators [13,30–32].

Carbon nanotubes are widely used for the fabrication of electrochemical biosensors, because of their special structure and properties such as high surface area, which makes them a suitable material to transfer electrons [33–35]. Gold nanoparticles are one of the most stable metal nanoparticles, and have been widely applied in analytical chemistry and electrochemistry, because of their novel optical, electrical and catalytic properties and favorable biocompatibility [36,37]. Each type of nano-material has its own physical and chemical characteristics, which makes the design and preparation of biosensors based on only one type of nanomaterial laborious. Thus, the use of composites from several type of materials could be preferable [38]. Recently, several nano materials, including carbon nanotubes and nanoparticles of Au, Ag, TiO_2_, Fe_3_O_4_ or MnO_2_, have been applied in electrochemical studies of hemoglobin and other redox proteins [39]. Immoblization of redox proteins on nanocomplexes is a new way to realize their direct electrochemistry.

In our previous study, a nanocomplex consisting of carboxylic acid functionalized multi-walled carbon nanotubes (FMWCNTs) and gold nanoparticles (AuPNs) was modified on a glassy carbon (GC) electrode, and applied to analysis the electrochemical properties of catalase [40] and heme-containing artificial peroxidase [41].

In the present study, direct electron transfer of Hb was realized when it was immobilized on the nanocomplex-modified glassy carbon (GC) electrode. Thus, this electrode could be used as a high sensitivity hydrogen peroxide (H_2_O_2_) biosensor.

## 2. Experimental Section

### 2.1. Chemicals

Hb from bovine erothrocyte, L-cysteine (Cys), NF (5%), HAuCl_4_ and sodium citrate were from Sigma (Saint Louis, MO, USA) and used without further purification. Multi-wall carbon nanotubes (MWCNTs), prepared by chemical vapor deposition, were purchased from Shenzhen Nanotech Port Ltd. Co. (Shenzhen, China). Hydrogen peroxide, sodium dihydrogen phosphate (NaH_2_PO_4_) and disodium hydrogen phosphate (Na_2_HPO_4_) were obtained from Shanghai Chemicals Company (Shanghai, China). All solutions were prepared in double-distilled deionized water. The stock solutions of hydrogen peroxide (H_2_O_2_) were prepared by appropriate dilutions of 30% (v/v) H_2_O_2_ in deionized water. All other chemicals were of analytical grade and used without further purification.

### 2.2. Preparation of Gold Nanoparticles (AuNPs)

AuNPs were prepared as previously reported in the literature [42–44]. Briefly, 0.01% HAuCl_4_ solution was heated, then 0.02 M sodium citrate solution was dropped quickly into the hot HAuCl_4_ solution while agitating vigorously. At first, the color of solution changed from light yellow to grey, then to black and gradually to wine red color without further change. At this stage, the color no longer changed, the heating was stopped, and the solution mixture was continuously agitated until it was cooled to room temperature. The prepared gold colloidal nanoparticles (AuNPs) were stored in dark at 4 °C.

### 2.3. Preparation of Functional Multi-Walled Carbon Nanotubes (FMWCNTs)

MWCNTs were functionalized according to published methods [23]. Briefly, purified MWCNTs were treated with a concentrated mixture of H_2_SO_4_ and HNO_3_ (v/v = 1/3) under supersonic bath condition (KQ-100B Supersonic Cleaner, Kunshan Shumei, Kunshan, China) for 4 h at 80 °C to introduce carboxyl groups on their surface. The solution pH was then adjusted to 7 with 1 M NaOH solution, centrifuged and washed with water three times. The obtained MWCNTs-COOH (FMWCNTs) were dried at room temperature.

### 2.4. Preparation of Functional Membrane Modified Glassy Carbon (GC) Electrode

**Figure 1.**
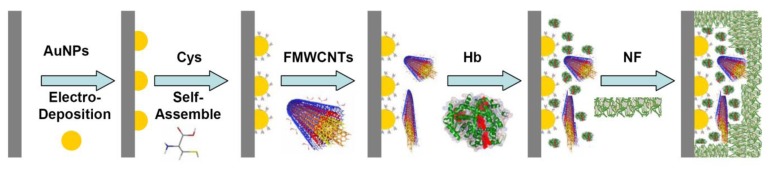
Preparation process of functional membrane modified glassy carbon (GC) electrode.

The preparation of the GC electrode was as previously described [45–49]. Prior to coating, the GC electrode was mechanically polished twice with alumina (particle sizes 1.00, 0.30 and 0.05 μm, respectively) to a mirror finish. The electrode was then treated electrochemically in 0.2 M sulfuric acid, cycling between −1.0 and +0.5 V (*vs.* Ag/AgCl) at a sweep rate of 0.1 V/s for approximately 10 min. Thereafter, the working electrode was placed in a 50 mM PBS (pH 7.0), and an anodic potential of 1.70 V (*vs.* Ag/AgCl) was applied for 3-5 min. After the electrode was washed, the AuNPs (negative charged) were electro-deposited on a cleaned bare GC electrode in the range of 0.0 to1.1 V 25 cycles at a scan rate of 0.1 V/s [50]. The GC electrode was then dipped in 1.0 mM L-cysteine (Cys) for 30 min, washed with water, 3 μL of FMWCNTs (2 mg/mL) was dropped onto the surface of the electrode, and dried at room temperature. The electrode was dipped in a Hb solution (80 μM) for 24 h at 4 °C, and for protection, 2 μL Nafion (NF, 5%) was dropped on the electrode surface. The preparation process of functional membrane modified glassy carbon (GC) electrode was also shown in Figure 1.

### 2.5. Apparatus and Measurements

Electrochemical studies were carried out in a conventional three-electrode cell powered by an electrochemical system comprising of CHI650C (CHI Instruments, Austin, TX, USA). An Ag/AgCl-saturated KCl, a Pt wire and a GC electrode of 3 mm diameter (CHI Instruments) were used as the reference, counter and working electrodes, respectively. All of the potentials in this article were with respect to Ag/AgCl. The electrochemical measurements were carried out in N_2_-saturated 0.05 M sodium phosphate buffer solution (PBS) at pH 7.0, 20 °C. Electron microscopic images (TEM) of FMWCNT and AuNPs were obtained using a JEM-1400 (JEOL, Musashino, Japan). Fourier transform infrared (FTIR) spectra of FMWCNTs by KBr pellets were cellected in the range of 1,000-3,500 cm^−1^ on a FTIR 4300 (Shimadzu, city, Japan) spectrometer at room temperature. UV-vis absorption spectra of the AuNPs were collected using a TU-1901 spectrophotometer (Beijing Purkinje General Instrument Company, Beijing, China), with 1 cm path length cells equipped with a thermostat holder and an external temperature controller (Shanghai Hengping Instrument Company, Shanghai, China) at 25 ± 0.1 °C.

## 3. Results and Discussion

### 3.1. Characteristics of the Nanomaterials

The AuPNs and FMWCNTs were characterized by TEM. 3 μL of AuPNs (0.254 mM) or FMWCNTs (3 mg/mL) was dropped onto the surface of fomvar/carbon coated grids (300 mesh), dried and then viewed by TEM operating at 80 kV [40], respectively. UV-vis spectroscopy of the prepared AuNPs exhibited a maximum absorption at 522 nm. The mean size of the AuNPs was then determined to be 18.4 ± 1.1 nm [40,51]. The FTIR spectrum of FMWCNTs shows the characteristic peaks at 1,709, 1,172, and 3,402 cm^1^ correspond to the C=O, C– O and O-H stretching vibration of the carboxyl group [52–54], respectively, which indicates that carboxyl groups were modified on the MWCNTs (data not shown).

### 3.2. Electrochemical Studies

**Figure 2.**
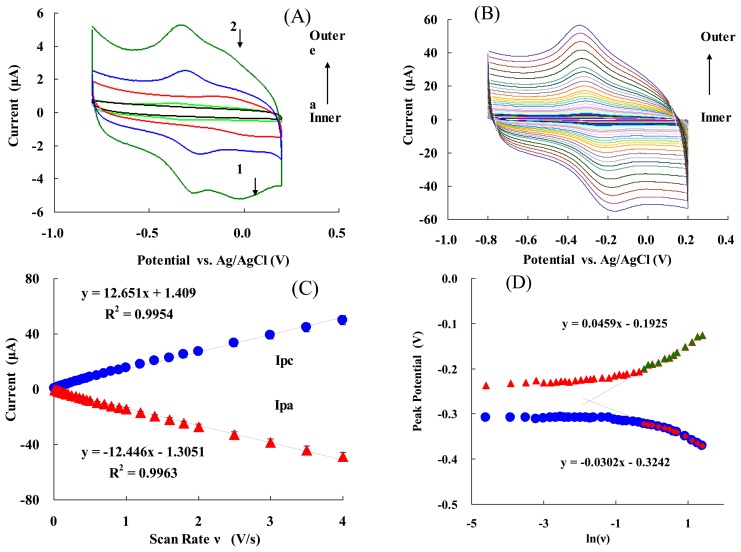
(**A**) CVs of different modified electrodes (from inner to outer): (a) Bare GC electrode; (b)NF/Hb/GC; (c) NF/FMWCNTs/Cys/AuNPs/GC; (d) NF/Hb/FMWCNTs/GC; (e) NF/Hb/FMWCNTs/Cys/AuNPs/GC. The experiments were carried out in 0.05 M PBS (pH7.0) at a scan rate of 0.05 V/s. (**B**) CVs of NF/Hb/FMWCNTs/Cys/AuNPs/GC electrode in 0.05 M PBS (pH 7.0) at various scan rates (from inner to outer): 0.02, 0.04, 0.06, 0.08, 0.1, 0.12, 0.14, 0.16, 0.18… 4 V/s, respectively; (**C**) Plot of peck current I_p_ *vs.* scan rate ν; (**D**) Plot of peak potential E_p_ *vs.* ln (ν).

Figure 2(A) presents the cyclic voltammograms (CVs) of: (a) bare electrode; (b) NF/Hb/GC electrode; (c) NF/FMWCNTs/Cys/AuNPs/GC electrode; (d) NF/Hb/FMWCNTs/GC electrode; and (e) NF/Hb/FMWCNTs/Cys/AuNPs/GC electrode at a scan rate of 0.05 V/s. It can be seen that either (d) or (e) show a well-defined redox wave. The electrode (e) had a stronger redox peak current than that of (d), and the AuPNs could help to significantly increase the redox peaks. Moreover, cathodic and anodic peak potentials were −0.309 V and −0.231 V (*vs.* Ag/AgCl), respectively. Thus, the formal potential (E°′ = (E_pa_ + E_pc_)/2) of the electrode (e) was−0.270 ± 0.002 V (*vs.* Ag/AgCl). This value is consistent with the E° ′ obtained for an Hb/PLGA/ILs/GC electrode (-0.318 V *vs.* SCE) [21], an Hb/BMS/CS/GC electrode (-0.32 V *vs.* Ag/AgCl) [55], or an Hb/Chit-[bmim]PF_6_-TiO_2_-Gr/GC electrode (-0.206 V *vs.* SCE) [17], which is the characteristics of the Hb heme Fe^(III)^/Fe^(II)^ redox couple. It is notable that the positive potential for the electrode facilitated the electrode reaction (Equation (1)) and led to a more efficient bio-catalytic reduction [46,47,49]:

(1) $$k_{s}={\text{Ae}}^{-\Delta G/\text{RT}}e^{-\alpha \text{nFE}}.$$

The separation between the anodic and cathodic peak potential (E_p_ = E_pa_ − E_pc_= 78 mV), and the current ratio of the anodic peak current to the cathodic one (I_pa_/I_pc_ ≈ 1), indicate that the electrochemical process of the modified GC electrode (e) is quasi-reversible [46,47]. Thus, AuNPs can greatly improve the redox current-modified electrode and facilitate a fast direct electron transfer of Hb entrapped in the composite film. In addition, it is worth noting that there was a pair of unstable redox peaks for curves at site 1 and 2, which should be due to Cys-modified Au, and this redox peak would disappear gradually while the electrode was working. CVs of NF/Hb/FMWCNTs/Cys/AuNPs/GC electrode in 50 mM PBS (pH 7.0) at various scan rates are shown in Figure 2(B). The peak currents increased with increasing the scan rate (ν) and were linearly proportional to ν (Figure 2C) (not ν^1/2^). The linear regression equations for cathodic (I_pc_) and anodic peak (I_pa_) currents are: I_pc_ = 12.65ν + 0.14 and I_pa_ =−12.45ν − 0.13, with the correlation coefficients of 0.9981 and 0.9977, respectively. The CVs remained essentially unchanged on consecutive potential cycling, indicating that modified electrode is stably confined on the glassy carbon electrode. Figure 2(D) shows the relationship between the peak potential (E_p_) and the natural logarithm of scan rate (lnν) for the modified electrode. In the range from 1.2 to 4 V/s, the cathodic peak potential (E_pc_) changed linearly *vs.* lnν with a linear regression equation of E_pc_ = − 0.0302 ln(ν) − 0.324, *r* = 0.991. According to Laviron's Equation [56]:

(2) $$E_{p}=E^{\circ \prime }+\frac{\text{RT}}{\alpha \text{nF}}-\frac{\text{RT}}{\alpha \text{nF}}\ln\upsilon$$

where *α* is the cathodic electron transfer coefficient, n is the number of electrons, T is the temperature (293 K here), R the gas constant (8.314 JK^−1^mol^−1^) and F the Faraday constant (96,485 C mol^−1^), respectively. RT/αnF was 0.0302 here, then, αn could be calculated to be 0.55 It could be concluded that *n* =1 and *α* = 0.55 ± 0.05 [57]. The *n* = 1 can be also obtained from the width of the peak at mid- height with a low scan rate. Thus, the redox reaction between Hb and the glassy carbon electrode is a single electron transfer process.

The value of the apparent heterogeneous electron transfer rate constant k_s_ could be calculated using the following equation based on Laviron's Equation [58]:

(3) $$\ln k_{s}=\alpha \ln(1-\alpha )+(1-\alpha )\ln\alpha -\ln(\frac{\text{RT}}{nF\nu })-\alpha (1-\alpha )\frac{\text{nF}\Delta E_{p}}{\text{RT}}.$$

Then, k_s_ was calculated to be 4.0 ± 0.2 s^−1^. This value was higher than the most reported k_s_ values of Hb immobilized on GC electrodes [5,9,10,17,23], due to the high specific surface area and good biocompatibility of the nano-complex.

The average surface concentration (Γ) of electro-active sites (heme groups) of Hb on the surface of glassy carbon electrode could be estimated based on the slope of I_p_ *vs.* ν (Equation (4)) [59]:

(4) $$I_{p}=\frac{n^{2}F^{2}A\Gamma \upsilon }{4\text{RT}}.$$

The value of Γ was calculated to be 6.8 ± 0.3 × 10^−10^ mol·cm^−2^, which is higher than the Γ value of monolayer of Hb 1.89 × 10^−11^ mol·cm^−2^[60,61]. This high surface concentration can be attributed to the AuNPs and FMWCNTs nanocomplex. The larger surface area and good biocompatibility of the nanocomplex may be helpful for more efficient Hb entrapped in the nanocomplex membrane and provides more activity sites to take part in the electron transfer process (see also Figure 1).

### 3.3. pH Effect

**Figure 3.**
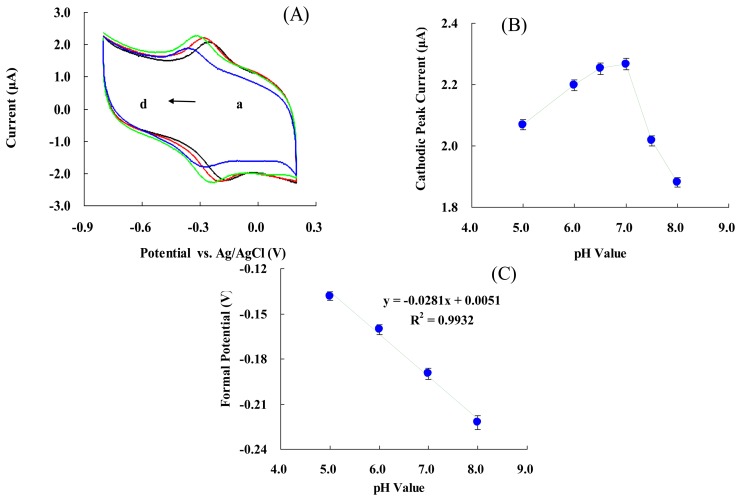
(**A**) CVs of NF/Hb/FMWCNTs/Cys/AuNPs/GC electrode in 0.05 M PBS at different pH values: (a) 5.0, (b) 6.0, (c) 7.0, and (d) 8.0, respectively; (**B**) plot of I_pc_ *vs.* pH value; (**C**) Plot of E ° ′ *vs.* pH value.

Figure 3(A) represents CVs of the modified electrode in 50 mM PBS at various pH values. An increase in pH of the solution from 5.0 to 8.0 led to a negative shift in both reduction and oxidation peak potentials. Figure 3(B) shows that the cathodic peak's current increased with pH changes from 5.0 to 7.0, and then decreased when the pH was greater than 7.0. The maximum cathodic current was obtained at pH 7.0. Figure 3(C) shows that the formal potential of the electrode is pH dependent. These results indicated that the slope was 28.1 ± 0.4 mV/pH over a pH range of 5.0 to 8.0. This value was much smaller than the ideal Nernst's value of 59.2 mV/pH for a one electron and one proton process [59]. The reason might be the biocompatible micro-environment provided by NF. This makes the electrode more stable to pH changes [45–48], influences the protonation state of *trans* ligands to the heme iron and amino acids around the heme, or the protonation of the water molecules coordinated to the central iron [62].

### 3.4. Optimum Monitoring Potential

**Figure 4.**
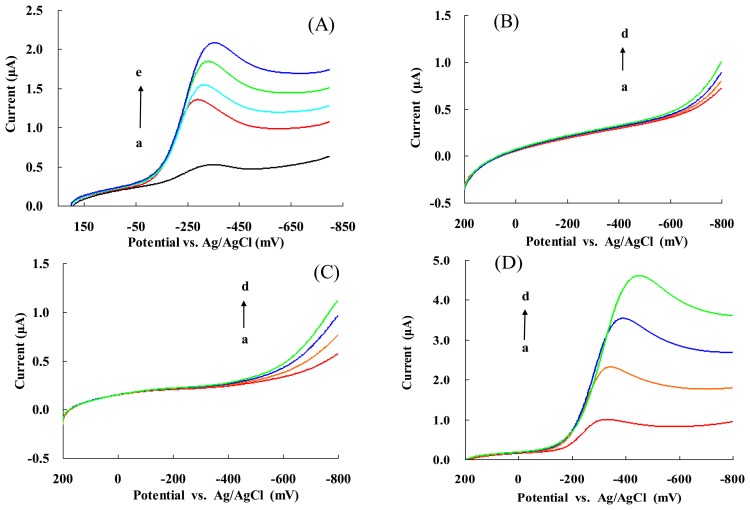
LSVs of (**A**) NF/Hb/FMWCNTs/Cys/AuNPs/GC electrode in the absence or presence of different concentrations of H_2_O_2_ (from curve a to curve e): 0, 0.1, 0.13, 0.16, 0.20 mM, respectively. LSVs of (**B**) Cys/AuNPs/GC; (**C**) NF/Cys/AuNPs/GC and (**D**) NF/Hb/Cys/AuNPs/GC electrodes, respectively in the presence of different concentrations of H_2_O_2_ (from a to curve d): 0.1, 0.3, 0.5 and 0.7 mM, respectively.

Figure 4(A) shows the linear sweep voltammograms (LSVs) of NF/Hb/FMWCNTs/Cys/AuNPs/GC electrode in the absence (a) and presence of H_2_O_2_ and with 0.1, 0.13, 0.16, 0.2 mM H_2_O_2_ (b-e). The cathodic peak current increased with increased concentration of H_2_O_2_. Thus, the electrode is sensitive to the tested concentrations of the chosen substrate and used as a biosensor of H_2_O_2_. In addition, the ΔI reached a maximum value for each concentration of H_2_O_2_ at about −350 mV. Hence, the potential of −350 mV (*vs.* Ag/AgCl) was selected as the optimized monitoring potential throughout this study.

It is worth noting that either Cys/AuNPs/GC (Figure 4(B)) or NF/Cys/AuNPs/GC (Figure 4(C)) electrodes exhibited an amperometric response to H_2_O_2_ at potentials higher than −0.6 V (*vs.* Ag/AgCl). These results were similar to those reported in the literature [63,64]. However, Figure 4(D) shows that the amperometric response of the NF/Hb/AuNPs/GC electrode toward H_2_O_2_ occurred at an even lower potential (-400 mV) with stronger current intensity. Thus, these results could be attributed to the bioelectro-catalytic behavior of Hb.

### 3.5. Electro-Catalytic Behavior of Modified Electrode and Detection Limit

**Figure 5.**
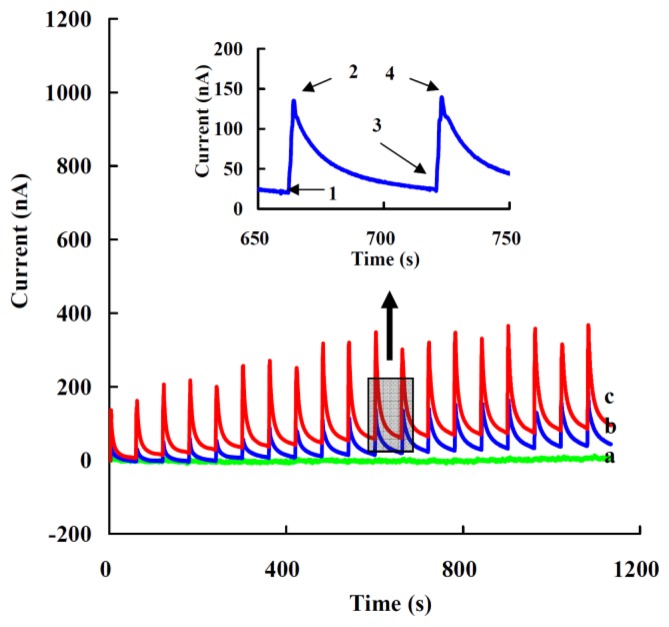
Amperometirc response of the modified electrode to successive additions of 5 μL of 1 nM (a), 10 nM(b) or 20 nM(c) H_2_O_2_ in 5 mL of 0.05 M PBS, pH 7.0, at the applied potential of −0.35 V *(vs.* Ag/AgCl). Inset shows the typical current response for each addition process: (1) previous steady state current; (2) maximum response current; (3) steady state current; (4). next maximum response current.

**Figure 6.**
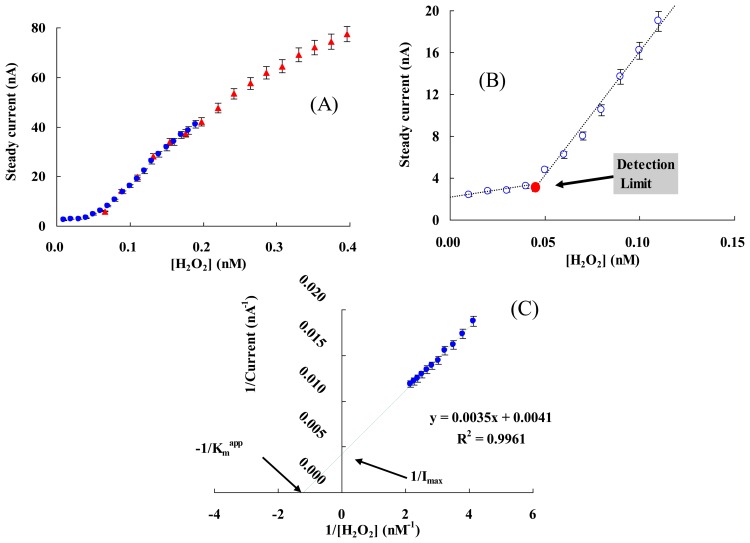
(**A**) The typical steady current *vs.* [H_2_O_2_] in the process of successive additions of 5 μL of 10 nM (●) and 20 nM (▲; H_2_O_2_ in 5 mL of 50 mM PBS (pH 7.0) at the applied potential of −0.35 V *(vs.* Ag/AgCl). (**B**) The determination of the H_2_O_2_ detection limit for NF/Hb/FMWCNTs/Cys/AuNPs/GC electrode. The detection limit was determined from the cross point of the lines fitted to the linear segments of the steady current Is *vs.* [H_2_O_2_] in the process of successive additions of 5 μL of 10 nM H_2_O_2_ in 5 mL of 50 mM PBS (pH7.0). (**C**) Lineweaver-Burk plot for K_m_^app^ determination.

Figure 5 presents the current responses of the modified electrode to successive additions of 5 μL of 1 nM (a), 10 nM (b) or 20 nM (c) of H_2_O_2_ in 5 mL of 0.05 M PBS (pH 7.0) at the applied potential of −0.35 V (*vs.* Ag/AgCl). The inset shows the typical current response for each addition process. Current at state 1 is a steady state (I_sa_) with no addition, when H_2_O_2_ is added the current increases rapidly and reaches a maximum, state 2 (I_ma_). The current then reduces gradually to a steady state, state 3 (I_sb_), before the next addition and next maximum current response (I_mb_), state 4. Though the amount of maximum response current was great, this state was unsuitable for use, as the change of the maximum current response (ΔI = I_mb_ − I_ma_) was not stable for each addition, and was affected by the mainly addition position and diffusion rate of H_2_O_2_. The steady state current response (ΔI = I_sb_ − I_sa_) increased when the addition concentration of added H_2_O_2_ was over 10 nM with the final concentration of H_2_O_2_ (0.01-50 nM) and a linear range from 0.05 to 1 nM (see also Figure 6(A)).

To determine the detection limit (minimum concentration of H_2_O_2_ that could be detected by this method), the steady current (I_s_) of each addition was measured while the final concentration of H_2_O_2_ ([H_2_O_2_]) was increased gradually (Figure 6(B)). The detection limit was determined to be 0.05 ± 0.01 nM from the cross point of the lines fitted to the linear segments of the I_s_ *vs.* [H_2_O_2_] [65,66], which was lower than the most reported values, e.g., an Hb/GNPs/Hb/MWNT/GC electrode (80 nM) [67] and a CAT/[bmim] [PF_6_]/MWCNTs/GC electrode (0.25 nM) [68].

### 3.6. Kinetic Parameters

Overall, the electrode reaction could be supposed to be carried out by two steps [40]:

$$\begin{array}{c} \text{Step One}:\text{Hb}(\text{F}{\text{e}}^{\text{III}})+\text{e}+{\text{H}}^{+}=\text{Hb}(\text{F}{\text{e}}^{\text{II}}-\text{H})\\ \text{Step Two}:\text{Hb}(\text{F}{\text{e}}^{\text{II}}-\text{H})+1/2{\text{H}}_{2}{\text{O}}_{2}\to \text{Hb}(\text{F}{\text{e}}^{\text{III}})+{\text{H}}_{2}\text{O} \end{array}$$

where, Hb(Fe^III^) and Hb(Fe^II^-H) denote the oxidized and reduced forms of the modified Hb, respectively. Initially Hb(Fe^III^) undergoes the electron transfer reaction with the electrode resulting in the production of Hb(Fe^II^-H), as shown in Step One, this is an one-electron and one-proton (pH dependent) process. Alternatively, Hb(Fe^II^-H) can be oxidized by H_2_O_2_ in the solution to regenerate Hb(Fe^III^) as shown in Step Two. In this catalytic reaction, the peak current kept rising until Hb(Fe^III^) was consumed in the Step One and was compensated by its production in Step Two.

The apparent Michaelis-Menten constant K_m_^app^ is a reflection of both enzyme affinity and ratio of microscopic kinetic constants. It can be obtained from the electrochemical version of the Linewearver-Burk Equation [13]:

(5) $$\frac{1}{I_{ss}}=\frac{1}{I_{\max}}+\frac{K_{m}^{\text{app}}}{I_{\max}c}$$

where, *c* is the substrate (H_2_O_2_) concentration in the solution ([H_2_O_2_] here), I_SS_ the steady-state current after the addition of substrate, and I_max_ is the maximum current measured under saturated substrate conditions. K_m_^app^ value of the modified electrode was calculated to be 0.85 ± 0.1 nM (Figure 6(C)), which was much lower than the most reported values, e.g., an HRP-AQ/GC electrode (51 nM) [49], a PLGA/ILs/Hb/GC electrode (69 μM) [21], and an Hb/Chit-[bmim]PF_6_-TiO_2_-Gr/GC electrode (1.245 mM) [17]. A low K_m_^app^ value indicates a strong substrate binding and exhibits a higher affinity of H_2_O_2_ for this modified electrode. FMWCNTs and AuNPs complex system helps to reduce the bridge length between electroactive center (heme group) of Hb and GC electrode [47], and result in the high sensitivity of the modified electrode to H_2_O_2_ (Table 1).

### 3.7. Stability

Long-term stability is an important parameter for biosensors. The operational stability of the modified electrode was determined by the CV method. The cathodic peak current was reduced by less than 5% after 50 cycles at the scan rate of 0.05 V/s, while the peak potential remained unchanged. As for the storage stability, the CVs showed minimal change after two weeks of storage in a bottle over the PBS solution at 4 °C. NF may offer a biocompatible micro-environment to confine bio-macromolecules at their ionic cluster region (30–50 nm), and this view was also consistent with our previous study [45–48]. Moreover, Nafion may be helpful to restrict, confirm and protect Hb/FMWCNTs/Cys/AuNPs system on GC electrodes.

### 3.8. Interference Determination

The degree of interference from interfering substances can be evaluated by the value of the cathodic current ratio which were calculated by reading the cathodic current (I_pc1_) of the proposed biosensor in 50 mM PBS (pH 7.0) containing 0.10 mM H_2_O_2_ and a 0.20 mM hampering substance, and then, comparing it with the cathodic current (I_pc0_) from the proposed biosensor in the same solution containing only 0.10 mM H_2_O_2_. Five interfering substances were tested here and the results are listed in Table 2. It can be observed that none of the tested interferents could cause interference to the determination of H_2_O_2_, which is largely attributed to the low working potential of −350 mV used in the determination of H_2_O_2_ and the negatively charged NF protection membrane.

## 4. Conclusions

The direct electrochemical properties of immobilized Hb on a FMWCNTs/Cys/AuNPs-modified glassy carbon electrode were found to be due to the excellent microenvironment provided by NF, AuNPs and FMWCNTs for Hb. The small value of K_m_^app^, high sensitivity, long-term stability and low detection limit were other characteristics of the biosensor. The modified electrode showed the ability to be used as a third generation biosensor for determination of H_2_O_2_ at ultra-trace levels. Moreover, a redox protein on the functional nano complex modified electrode may be a new useful electrochemical tool for the analysis of relationship between the structure and function of redox proteins, especially for a heme-containing protein.

## Figures and Tables

**Table 1.** Comparison of electrochemical parameters of Hb on different modified GC electrodes.

**Modified Electrode**	**E°′** (mV)	**k_s_** (s^−1^)	**Γ (mol·cm^−2^)**	**K_m_^app^**	**Linear Range**	**Detection Limit**	**Ref**
NF/Hb/FMWCNTs/Cys/AuNPs/GC	−270 ± 2 a	4.0 ± 0.2	6.8 ± 0.3 × 10^−10^	0.85 nM	0.05–1 nM	0.05 nM	This work
Hb/PdNPs/GR-CS/GC	−240 b	0.69	1.74 × 10^−10^	16 μM	2–1100 μM	660 nM	[5]
Hb/NiO/GC	−70 a	5.2 ± 0.5	1.73 × 10^−11^	1370 μM	1–2000 μM	630 nM	[6]
NF/Hb/PAM-P123/GC	−317 b	-	7.64 × 10^−11^	36 μM	1–30 μM	400 nM	[7]
Hb/Gel/GC	−380 b	-	-	-	50–1200 μM	3400 μM	[8]
Hb/NGC-SF/GC	−380 b	1.98	-	-	0.6–1.7 and 2–22 mM	-	[9]
Polymer-Hb-CNTs/GC	−273 b	0.90	1.1 × 10^−10^	140 μM	8–240 μM	4 μM	[10]
Hb/CS-[bmim] PF_6_-TiO_2_-GR/GC	−206 b	0.73-3.96	3.21 × 10^−10^	1245 μM	1–1170 μM	0.3 μM	[17]
Hb/PLGA/ILs/GC	−318 b	5.02 ± 0.16	4.74 × 10^−10^	69 μM	5–8050 μM	0.237 μM	[21]
Hb/ATP/GC	−362 b	4.6 ± 0.65	6.7 × 10^−11^	490 μM	5.4–400 μM	2.4 μM	[22]
EDC-Hb-CNTs/GC	−268 b	1.02 ± 0.05	4.7 × 10^−9^	-	0.25–140 μM	0.18 μM	[23]
Hb/BMS/CS/GC	−320 a	-	9.34 × 10^−11^	-	2.5–245 μM	0.83 μM	[55]
Hb/GNPs/Hb/MWNT/GC	−355 b	-	-	260 μM	0.21–3000 μM	80 nM	[67]

NF: Nafion; GR: graphene; PdNPs: palladium nanoparticles; CS: chitosan; NiO: nickel oxide nanoparticles; PLGA: poly lactic-co-glycolic acid; ILs: ionic liquid, 1-butyl-3-methylimidazolium tetrafluoroborate ([BMIM]BF4; BMS: bimodal mesoporous silica; PAM-P123: polyacrylamide-P123; Gel: gelatine; NGC: nanostructured gold colloid; SF: silk fibroin; EDC: 1-ethyl-3-(3-dimethylaminopropyl) carbodiimide; ATP: attapulgite; GNPs: gold colloidal nanoparticles

a *vs.* Ag/AgCl;

b *vs.* SCE.

**Table 2.** Effects of possible interferences on the hydrogen peroxide biosensor.

**Possible Interferences**	**Ipc1/Ipc0**	**R.S.D (%)**
Glucose	1.04 ± 0.08	3.2
Adenosine Triphosphate	1.03 ± 0.06	3.4
L-Histidine	1.01 ± 0.05	2.9
Ascorbic Acid	0.98 ± 0.06	3.1
Thiol	1.00 ± 0.04	2.5

**I_pc1_**: The cathodic current for a mixture of a 0.20 mM interfering substance and 0.10 mM H_2_O_2_; **I_pc0_**: The cathodic current for 0.10 mM H_2_O_2_ alone in a 50 mM PBS (pH 7.0), at −350 mV *vs.* Ag/AgCl. R.S.D: Relative standard deviation, obtained for nine measurements.

## References

[1] Armstrong F.A., Hill H.A.O., Walton N.J. (1988). Direct electrochemistry of redox proteins. Acc. Chem. Res..

[2] Armstrong F.A., Wilson G.S. (2000). Recent developments in faradaic bioelectrochemistry. Electrochim. Acta.

[3] Hill H.A.O. (1996). Direct electrochemistry of cytochrome c. Coord. Chem. Rev..

[4] Thévenot D.R., Toth K., Durst R.A., Wilson G.S. (2001). Electrochemical biosensors: Recommended definitions and classification. Biosens. Bioelectron..

[5] Sun A., Sheng Q., Zheng J. (2012). A hydrogen peroxide biosensor based on direct electrochemistry of hemoglobin in palladium nanoparticles/graphene–chitosan nanocomposite film. Appl. Biochem. Biotechnol..

[6] Salimi A., Sharifi E., Noorbakhsh A., Soltanian S. (2006). Direct voltammetry and electrocatalytic properties of hemoglobin immobilized on a glassy carbon electrode modified with nickel oxide nanoparticles. Electrochem. Commun..

[7] Li J., Tang J., Zhou L., Han X., Liu H. (2012). Direct electrochemistry and electrocatalysis of hemoglobin immobilized on polyacrylamide-P123 film modified glassy carbon electrode. Bioelectrochemistry.

[8] Yao H., Li N., Xu J.Z., Zhu J.J. (2007). Direct electrochemistry and electrocatalysis of hemoglobin in gelatine film modified glassy carbon electrode. Talanta.

[9] Guo H.L., Liu D.Y., Yu X.D., Xia X.H. (2009). Direct electrochemistry and electrocatalysis of hemoglobin on nanostructured gold colloid-silk fibroin modified glassy carbon electrode. Sens. Actuators B Chem..

[10] Chen L., Lu G. (2006). Direct electrochemistry and electrocatalysis of hybrid film assembled by polyelectrolyte–surfactant polymer, carbon nanotubes and hemoglobin. J. Electroanal. Chem..

[11] Lee K.P., Gopalan A.I., Komathi S. (2009). Direct electrochemistry of cytochrome c and biosensing for hydrogen peroxide on polyaniline grafted multi-walled carbon nanotube electrode. Sens. Actuators B Chem..

[12] Liu X.J., Zhang W.J., Huang Y.X., Li G.X. (2004). Enhanced electron-transfer reactivity of horseradish peroxidase in phosphatidylcholine films and its catalysis to nitric oxide. J. Biotechnol..

[13] Liu Y., Han T., Chen C., Bao N., Yu C.M., Gu H.Y. (2011). A novel platform of hemoglobin on core-shell structurally Fe3O4@Au nanoparticles and its direct electrochemistry. Electrochim. Acta.

[14] Rhieu S.Y., Ludwig D.R., Siu V.S., Palmore G.T.R. (2009). Direct electrochemistry of cytochrome P450 27B1 in surfactant films. Electrochem. Commun..

[15] Rusling J.F., Nassar A.E.F. (1993). Enhanced electron transfer for myoglobin in surfactant films on electrodes. J. Am. Chem. Soc..

[16] Shang L.B., Sun Z.Y., Wang X.W., Li G.X. (2003). Enhanced peroxidase activity of hemoglobin in a DNA membrane and its application to an unmediated hydrogen peroxide biosensor. Anal. Sci..

[17] Sun J.Y., Huang K.J., Zhao S.F., Fan Y., Wu Z.W. (2011). Direct electrochemistry and electrocatalysis of hemoglobin on chitosan-room temperature ionic liquid-TiO_2_-graphene nanocomposite film modified electrode. Bioelectrochemistry.

[18] Xu J.S., Zhao G.C. (2008). A third-generation biosensor based on the enzyme-like activity of cytochrome c on a room temperature ionic liquid and gold nanoparticles composite. Int. J. Electrochem. Sci..

[19] Yang J., Hu N. (1999). Direct electron transfer for hemoglobin in biomembrane-like dimyristoyl phosphatidylcholine films on pyrolytic graphite electrodes. Bioelectrochem. Bioenerg..

[20] Yang N., Hoffmann R., Smirnov W., Kriele A., Nebel C.E. (2010). Direct electrochemistry of cytochrome c on nanotextured diamond surface. Electrochem. Commun..

[21] Zhang Y., Sun X., Jia N. (2011). Direct electrochemistry and electrocatalysis of hemoglobin immobilized into poly (lactic-co-glycolic acid)/room temperature ionic liquid composite film. Sens. Actuators B Chem..

[22] Xu J., Li W., Yin Q., Zhong H., Zhu Y., Jin L. (2007). Direct electron transfer and bioelectrocatalysis of hemoglobin on nano-structural attapulgite clay-modified glassy carbon electrode. J. Colloid Interface Sci..

[23] Zhang R., Wang X., Shiu K.K. (2007). Accelerated direct electrochemistry of hemoglobin based on hemoglobin–carbon nanotube (Hb–CNT) assembly. J. Colloid Interface Sci..

[24] Gorton L., Lindgren A., Larsson T., Munteanu F.D., Ruzgas T., Gazaryan I. (1999). Direct electron transfer between heme-containing enzymes and electrodes as basis for third generation biosensors. Anal. Chim. Acta.

[25] Olsson M.G., Allhorn M., Olofsson T., AKerstrom B. (2007). Up-regulation of alpha1-microglobulin by hemoglobin and reactive oxygen species in hepatoma and blood cell lines. Free Radic. Biol. Med..

[26] Buehler P.W., Haney C.R., Gulati A., Ma L., Hsia C.J.C. (2004). Polynitroxyl hemoglobin: A pharmacokinetic study of covalently bound nitroxides to hemoglobin platforms. Free Radic. Biol. Med..

[27] Royer W.E., Knapp J.E., Strand K., Heaslet H.A. (2001). Cooperative hemoglobins: Conserved fold, diverse quaternary assemblies and allosteric mechanisms. Trends Biochem. Sci..

[28] Ding Y., Wang Y., Li B.K., Lei Y. (2010). Electrospun hemoglobin microbelts based biosensor for sensitive detection of hydrogen peroxide and nitrite. Biosens. Bioelectron..

[29] He X.Y., Zhu L. (2006). Direct electrochemistry of hemoglobin in cetylpyridinium bromide film: Redox thermodynamics and electrocatalysis to nitric oxide. Electrochem. Commun..

[30] Ferapontova E.E., Gorton L. (2005). Direct electrochemistry of heme multicofactor-containing enzymes on alkanethiol-modified gold electrodes. Bioelectrochemistry.

[31] Parak F.G., Nienhaus G.U. (2002). Myoglobin, a paradigm in the study of protein dynamics. Chemphyschem.

[32] Cao W.X., Christian J.F., Champion P.M., Rosca F., Sage J.T. (2001). Water penetration and binding to ferric myoglobin. Biochemistry.

[33] Ajayan P.M. (1999). Nanotubes from carbon. Chem. Rev..

[34] Ebbesen T.W., Ajayan P.M. (1992). Large-Scale synthesis of carbon nanotubes. Nature.

[35] Iijima S. (1991). Helical microtubules of graphitic carbon. Nature.

[36] Chen H.J., Wang Y.L., Wang Y.Z.H., Dong S.H.J., Wang E.K. (2006). One-Step preparation and characterization of PDDA-protected gold nanoparticles. Polymer.

[37] Nada M.D., David M.B. (2001). Radiolytically induced formation and optical absorption spectra of colloidal silver nanoparticles in supercritical ethane. J. Phys. Chem. B.

[38] Chen G.F., Liang Z.Q., Li G.X. (2010). Progress of electrochemical biosensors fabricated with nanomaterials. Acta Biophys. Sin..

[39] Scheller F.W., Bistolas N., Liu S.Q., Jänchen M., Katterle M., Wollenberger U. (2005). Thirty years of haemoglobin electrochemistry. Adv. Colloid Interface Sci..

[40] Hong J., Yang W.Y., Zhao Y.X., Xiao B.L., Gao Y.F., Yang T., Ghourchian H., Moosavi-Movahedi Z., Sheibani N., Li J.G. (2013). Catalase immobilized on a functionalized multi-walled carbon nanotubes–gold nanocomposite as a highly sensitive bio-sensing system for detection of hydrogen peroxide. Electrochim. Acta.

[41] Yang W.Y., Hong H., Zhao Y.X., Xiao B.L., Gao Y.F., Yang T., Moosavi-Movahedi A.A., Ghourchian H., Moosavi-Movahedi Z. (2013). Electrochemical study of a nano vesicular artificial peroxidase on a functional nano complex modified glassy carbon electrode. J. New Mat. Electrochem. Syst..

[42] Lin S.Q., Ju H.X. (2002). Renewable reagentless hydrogen peroxide sensor based on direct electron transfer of horseradish peroxidase immobilized on colloidal gold-modified electrode. Anal. Biochem..

[43] Daniel M.C., Astruc D. (2004). Gold Nanoparticles: Assembly, supramolecular chemistry, quantum-Size-related properties, and applications toward biology, catalysis, and nanotechnology. Chem. Rev..

[44] Xian Y., Hu Y., Liu F., Xian Y., Wang H., Jin L. (2006). Glucose biosensor based on Au nanoparticles–conductive polyaniline nanocomposite. Biosens. Bioelectron..

[45] Hong J., Ghourchian H., Moosavi-Movahedi A.A. (2006). Direct electron transfer of redox proteins on a Nafion-cysteine modified gold electrode. Electrochem. Commun..

[46] Hong J., Ghourchian H., Rezaei-zarchi S., Moosavi-Movahedi A.A., Ahmadian S., Saboury A.A. (2007). Nafion-methylene blue functional memberane and its application in chemical/bio-sensing. Anal. Lett..

[47] Hong J., Moosavi-Movahedi A.A., Ghourchian H., Molaei Rad A. (2007). Direct electron transfer of horseradish peroxidase on Nafion-cysteine modified gold electrode. Electrochim. Acta.

[48] Rezaei-Zarchi S., Saboury A.A., Hong J., Norouzi P., Moghaddam A.B., Ghourchian H., Ganjali M.R., Moosavi-Movahedi A.A., Javed A., Mohammadian A. (2007). Electrochemical behavior of redox proteins immobilized on Nafion-riboflavin modified gold electrode. Bull. Korean Chem. Soc..

[49] Shourian M., Ghourchian H. (2010). Biosensing improvement of horseradish peroxidase towards hydrogen peroxide upon modifying the accessible lysines. Sens. Actuators B Chem..

[50] Tian Y., Mao L., Okajima T., Ohsaka T. (2005). A carbon fiber microelectrode-based third-generation biosensor for superoxide anion. Biosens. Bioelectron..

[51] Haiss W., Thanh N.T.K., Aveyard J., Fernig D.G. (2007). Determination of size and concentration of gold nanoparticles from UV-vis spectra. Anal. Chem..

[52] Park M.J., Lee J.K., Lee B.S., Lee Y.W., Choi I.S., Lee S.G. (2006). Covalent modification of multiwalled carbon nanotubes with imidazolium-based ionic liquids: Effect of anions on solubility. Chem. Mater..

[53] Shen J., Huang W., Wu L., Hu Y., Ye M. (2007). Study on amino-functionalized multiwalled carbon nanotubes. Mater. Sci. Eng. A.

[54] Wang J., Fang Z., Gu A., Xu L., Liu F. (2006). Effect of amino-functionalization of multi-walled carbon nanotubes on the dispersion with epoxy resin matrix. J. Appl. Polym. Sci..

[55] Zhang L., Zhang Q., Li J. (2007). Direct electrochemistry and electrocatalysis of hemoglobin immobilized in bimodal mesoporous silica and chitosan inorganic–organic hybrid film. Electrochem. Commun..

[56] Laviron E.J. (1974). Adsorption, autoinhibition and autocatalysis in polarography and in linear potential sweep voltammetry. J. Electroanal. Chem..

[57] Ma H., Hu N., Rusling J.F. (2000). Electroactive myoglobin films grown layer-by-layer with poly(styrenesulfonate) on pyrolytic graphite electrodes. Langmuir.

[58] Laviron E.J. (1979). General expression of the linear potential sweep voltammogram in the case of diffusionless electrochemical systems. J. Electroanal. Chem..

[59] Rahimi P., Rafiee-Pour H., Ghourchian H., Norouzi P., Ganjali M.R. (2010). Ionic-liquid/NH2-MWCNTs as a highly sensitive nano-composite for catalase direct electrochemistry. Biosen. Bioelectron..

[60] Wang S., Chen T., Zhang Z., Shen X., Lu Z., Pang D., Wong K. (2005). Direct electrochemistry and electrocatalysis of heme proteins entrapped in agarose hydrogel films in room-temperature ionic liquids. Langmuir.

[61] Wang S., Chen T., Zhang Z., Pang D., Wong K. (2007). Effects of hydrophilic room-temperature ionic liquid 1-butyl-3-methylimidazolium tetrafluoroborate on direct electrochemistry and bioelectrocatalysis of heme proteins entrapped in agarose hydrogel films. Electrochem. Commun..

[62] Yamazaki I., Araiso T., Hayashi Y., Yamada H., Makino R. (1978). Analysis of acid-base properties of peroxidase and myoglobin. Adv. Biophys..

[63] Wang L., Bo X., Bai J., Zhu L., Guo L. (2010). Gold nanoparticles electrodeposited on ordered mesoporous carbon as an enhanced material for nonenzymatic hydrogen peroxide sensor. Electroanalysis.

[64] Jirkovsky J.S., Halasa M., Schiffrin D.J. (2010). Kinetics of electrocatalytic reduction of oxygen and hydrogen peroxide on dispersed gold nanoparticles. Phys. Chem. Chem. Phys..

[65] Molaei Rad A., Ghourchian H., Moosavi-Movahedi A.A., Hong J., Nazari K. (2007). Spectrophotometric assay for horseradish peroxidase activity based on pyrocatechol–aniline coupling hydrogen donor. Anal. Biochem..

[66] Buck R.P., Linder E. (1994). Recommendations for nomenclature of ion selective electrodes. Pure Appl. Chem..

[67] Chen S., Yuan R., Chai Y., Zhang L., Wang N., Li X. (2007). Amperometric third-generation hydrogen peroxide biosensor based on the immobilization of hemoglobin on multiwall carbon nanotubes and gold colloidal nanoparticles. Biosens. Bioelectron..

[68] Shamsipur M., Asgari M., Maragheh M.G., Moosavi-Movahedi A.A. (2012). A novel impedimetric nanobiosensor for low level determination of hydrogen peroxide based on biocatalysis of catalase. Bioelectrochemistry.

